# Single point mutations at the S129 residue of α-synuclein and their effect on structure, aggregation, and neurotoxicity

**DOI:** 10.3389/fchem.2023.1145877

**Published:** 2023-05-26

**Authors:** Esha Pandit, Lopamudra Das, Anoy Kumar Das, Sandip Dolui, Saumen Saha, Uttam Pal, Animesh Mondal, Joydeep Chowdhury, Subhas C. Biswas, Nakul C. Maiti

**Affiliations:** ^1^ Structural Biology and Bioinformatics Division, Indian Institute of Chemical Biology, Council of Scientific and Industrial Research, Kolkata, India; ^2^ Cell Biology and Physiology Division, CSIR-Indian Institute of Chemical Biology, Kolkata, India; ^3^ Department of Physics, Jadavpur University, Kolkata, India

**Keywords:** amyloid, fibrillization, secondary structure, alpha-synuclein, neurotoxicity, Raman

## Abstract

Parkinson’s disease is an age-related neurological disorder, and the pathology of the disease is linked to different types of aggregates of α-synuclein or alpha-synuclein (aS), which is an intrinsically disordered protein. The C-terminal domain (residues 96–140) of the protein is highly fluctuating and possesses random/disordered coil conformation. Thus, the region plays a significant role in the protein’s solubility and stability by an interaction with other parts of the protein. In the current investigation, we examined the structure and aggregation behavior of two artificial single point mutations at a C-terminal residue at position 129 that represent a serine residue in the wild-type human aS (wt aS). Circular Dichroism (CD) and Raman spectroscopy were performed to analyse the secondary structure of the mutated proteins and compare it to the wt aS. Thioflavin T assay and atomic force microscopy imaging helped in understanding the aggregation kinetics and type of aggregates formed. Finally, the cytotoxicity assay gave an idea about the toxicity of the aggregates formed at different stages of incubation due to mutations. Compared to wt aS, the mutants S129A and S129W imparted structural stability and showed enhanced propensity toward the α-helical secondary structure. CD analysis showed proclivity of the mutant proteins toward α-helical conformation. The enhancement of α-helical propensity lengthened the lag phase of fibril formation. The growth rate of β-sheet-rich fibrillation was also reduced. Cytotoxicity tests on SH-SY5Y neuronal cell lines established that the S129A and S129W mutants and their aggregates were potentially less toxic than wt aS. The average survivability rate was ∼40% for cells treated with oligomers (presumably formed after 24 h of incubation of the freshly prepared monomeric protein solution) produced from wt aS and ∼80% for cells treated with oligomers obtained from mutant proteins. The relative structural stability with α-helical propensity of the mutants could be a plausible reason for their slow rate of oligomerization and fibrillation, and this was also the possible reason for reduced toxicity to neuronal cells.

## Introduction

Parkinson’s disease (PD) is one of the most prevalent neurodegenerative disorders, and it affects the nervous system that controls the movement of human body parts. Typical clinical features of the diseases comprise tremor, muscle rigidity, dysarthria, dysphagia, and impaired balance (Schapira et al., 2017). The pathological hallmark of PD includes progressive loss of nigrostriatal dopamine neurons, swollen dystrophic neurites, and characteristic amyloid inclusions, called Lewy bodies (Xu et al., 2016). The filamentous intracellular inclusions are protein aggregates found in other neural disorders like dementia and multiple system atrophy (MSA) (Aniszewska et al., 2022). Along with proteins like p62 and ubiquitin, the chief component of the Lewy bodies is found to be α-synuclein (aS) (Spillantini et al., 1997). aS also contributes to the fibrillization of amyloid-β and tau, two key proteins linked to Alzheimer’s disease (AD) (Guo et al., 2013).

aS is a water soluble protein and largely found in the brain (Burré, 2022). It is a member of the synuclein family which also includes other proteins like β- and γ-synuclein (Kuusisto et al., 2003). This protein is abundantly expressed in the nervous system and is often localized at the presynaptic nerve terminals (Hawk et al., 2019). Even though the biological role and function of this protein are still an enigma, it is believed that the protein has a chaperone-like function and control release of neurotransmitters and it has effects on the SNARE complex (Maroteaux et al., 1988; Burré et al., 2010; Wang et al., 2018). aS consists of 140 amino acid residues; however, it does not have a defined globular and well folded structure. It thus belongs to the intrinsically disordered protein (IDP) family (Alderson and Markley, 2013). Earlier reports suggest that the protein is present in an unfolded monomeric confirmation in the cytosol and may have a stable α-helical conformation when bound to phospholipids (Burré et al., 2014). The protein has three distinct regions with a defined role in the protein structure and functions (Marvian et al., 2020). The positively charged N-terminal domain (residues 1–60) includes seven series of 11 amino acid (AA) repeats; each repeat contains a highly conserved KTKEGV hexamer motif (Bartels et al., 2010). These are similar to repeats present in the highly conserved α-helical domain of apolipoproteins. These repeats play a role in the lipid–protein interaction (Romero et al., 1997). The core region (residues 61–95), also known as the NAC (non-Aβ-amyloid component) domain, can form a cross β-sheet structure and is significantly involved in the aggregation process and fibril event of this highly soluble protein (Fields et al., 2019).

The C-terminal domain (residues 96–140) is the acidic tail of the protein, with low hydrophobicity and a high net negative charge, and possesses random and highly fluctuating conformation (Das et al., 2014; Schapira et al., 2017). The region contains binding sites that are involved in protein–protein interactions and a common binding interface for metal ions (Pal et al., 2016a). Some residues in this region are also much prone to post translational modifications (PTMs) (Schmid et al., 2013; Burré et al., 2018; González et al., 2019; Zhang et al., 2019; Liu et al., 2020). It is reported that the C-terminal domain has similarities with the α-crystalline domain of small heat shock proteins (Kim et al., 2004), which indicates its protective role in keeping the protein out of the degradation process (McLean et al., 2002; Cox et al., 2018; Jia et al., 2019). This region interacts with the NAC domain of the protein to inhibit and prevent its aggregation (Emamzadeh, 2016; Stephens et al., 2020). Moreover, calcium binding in this region modulates the protein interaction with synaptic vesicles (Lautenschläger et al., 2018). aS thus maintains a unique stability between its folded patches (rich with residues prone to aggregate, non-Aβ-amyloid component (NAC)) and highly disordered C-terminal regions in the solution state at physiological pH. The presence of such dynamic and flexible disordered regions in the protein may confer suitable plasticity to interact efficiently with several targets inside the cellular system. However, certain changes in the microenvironment cause misfolding of the protein, and it becomes the starting point of the aggregation process (Pal et al., 2016b). *In vitro* measurements also found that the mutations modulate protein aggregation and amyloid formation to a significant extent. For instance, A30P aggravates oligomerization, whereas E46K completely alters the fiber structure. Single point mutation or monogenic disorder is not very uncommon. Sickle cell anemia is a good example of such a case that leads to aggregation of hemoglobin, and it is due to a substitution of valine for glutamic acid in the structure of the β-chain hemoglobin molecule. It has also been demonstrated that the change in the chirality of an amino acid from L to D influences the aggregation kinetics (Chandra et al., 2017). In addition, for aS, several point mutations such as A18T, A29S, A30P, E46K, H50Q, G51D, A53E, and A53T are identified and it was observed that the rate of aggregation varies in each case (Appel-Cresswell et al., 2013; Lázaro et al., 2014; Flagmeier et al., 2016). For example, A30P greatly accelerates aS fibrillation, whereas E46K alters the core structure of the fibril (Chartier-Harlin et al., 2004). Interestingly, most of these mutations are present in the N-terminal end of the protein. The mutation in the C-terminal of the protein can also lead to a change in the protein–protein interaction, which may further affect the protein stability, change in conformation, and overall aggregation processes.

Our investigation was focused on the serine residue at position 129 (S129), which is found to be highly phosphorylated (89%) in aS present (Okochi et al., 2000; Fujiwara et al., 2002; Takahashi et al., 2003; Anderson et al., 2006) in the Lewy bodies. However, only 5% of the same residue was phosphorylated in the healthy brain samples. This phosphorylation, thus, assumed to have a significant effect on the aS aggregation pattern and subcellular distribution of the protein (Gonçalves and Outeiro, 2013; Pinho et al., 2019). Therefore, several investigations were carried out with S129A mutant protein (that prevents phosphorylation due to the lack of the serine residue at this position) to examine the role of phosphorylation on aggregation and toxic behavior to neuronal cells (Smith et al., 2005; McFarland et al., 2009; Lázaro et al., 2014; Oueslati, 2016; Ghanem et al., 2022). A study on the SH-SY5Y cell line by Smith et al. (2005), however, on the contrary, showed formation of less inclusion bodies inside the cell in the case of S129A mutation (Gasteiger et al., 2005). Thus, the study indicated that replacement of the residue with alanine reduced the aggregation behavior of the protein. However, what structural changes the S129A mutation brings and how that affects aggregation behavior of the protein were not known. Our interest was focused on deriving the structural information on the mutant protein and correlating this information with aggregation behavior of the protein in this altered situation. Thus, it gave us the opportunity to explore the role of this unique residue and associated structure in aggregation behavior of aS in an ambient solution condition. We prepared two recombinant artificial single point mutants of aS: S129W and S129A and investigated the protein secondary structure of the mutant proteins by CD and Raman spectroscopic methods. Furthermore, our investigation correlated the aggregation behavior of the protein with the alteration of the protein secondary structure in two mutant proteins. The derived results were compared to wt aS. In S129A, essentially, the polar side chain is replaced with a nonpolar methyl group and it was found that it drastically reduces the aggregation propensity of α-synuclein. Insertion of tryptophan residue in this position also produced interesting results. We further investigated the toxic effect on neuronal (SH-SY5Y) cell lines. The study established that increase in α-helicity in the secondary structure of the mutant proteins slowed down the aggregation and caused less damage to neuronal cells.

## Results and discussion

### Stability and their spectroscopic signatures of α-synuclein mutants

Established protocols were used to prepare and purify both the wild (wt aS) and mutant (S129A and S129W) human aS (Luth et al., 2015). The purity of the prepared protein solution in aqueous buffer was judged based on SDS-PAGE and mass spectroscopy analyses (Supplementary Figures S1A, S1B). Both the data confirmed the mass of the three proteins was ∼14 KDa. Absorbance and fluorescence spectra of freshly prepared protein solutions were recorded (Supplementary Figures S2A, S2B). The absorbance band appeared at ∼276 nm for wt aS and both the mutant proteins (Supplementary Figure S2A). Supplementary Figures S1B shows the fluorescence spectra of aqueous protein solutions at ambient conditions (20 mM phosphate buffer solution, pH 7.4). The wt aS and S129A showed fluorescence emission maxima at 302 nm, while the S129W mutant showed the peak at 356 nm. Lack of Trp residue in wild aS and S129A variant was indicated by very weak fluorescence at 302 nm due to four tyrosine residues present in the protein backbone. However, the strong fluorescence peak at 356 nm for S129W was caused due to substituted tryptophan. The emission Trp fluorescence is very sensitive to the polarity of the surrounding microenvironment of the tryptophan residue. Solvent-exposed Trp residues exhibit fluorescence peaks at around 350 nm; however, in the hydrophobic cavity of folded protein, the residue yields fluorescence at ∼342 nm (Vivian and Callis, 2001). The fluorescence peak position at 356 nm indicated that the tryptophan was well exposed to the solvent environment in the mutant protein, S129W.

### Secondary structural changes during aggregation by circular dichroism spectroscopy

**FIGURE 1:**
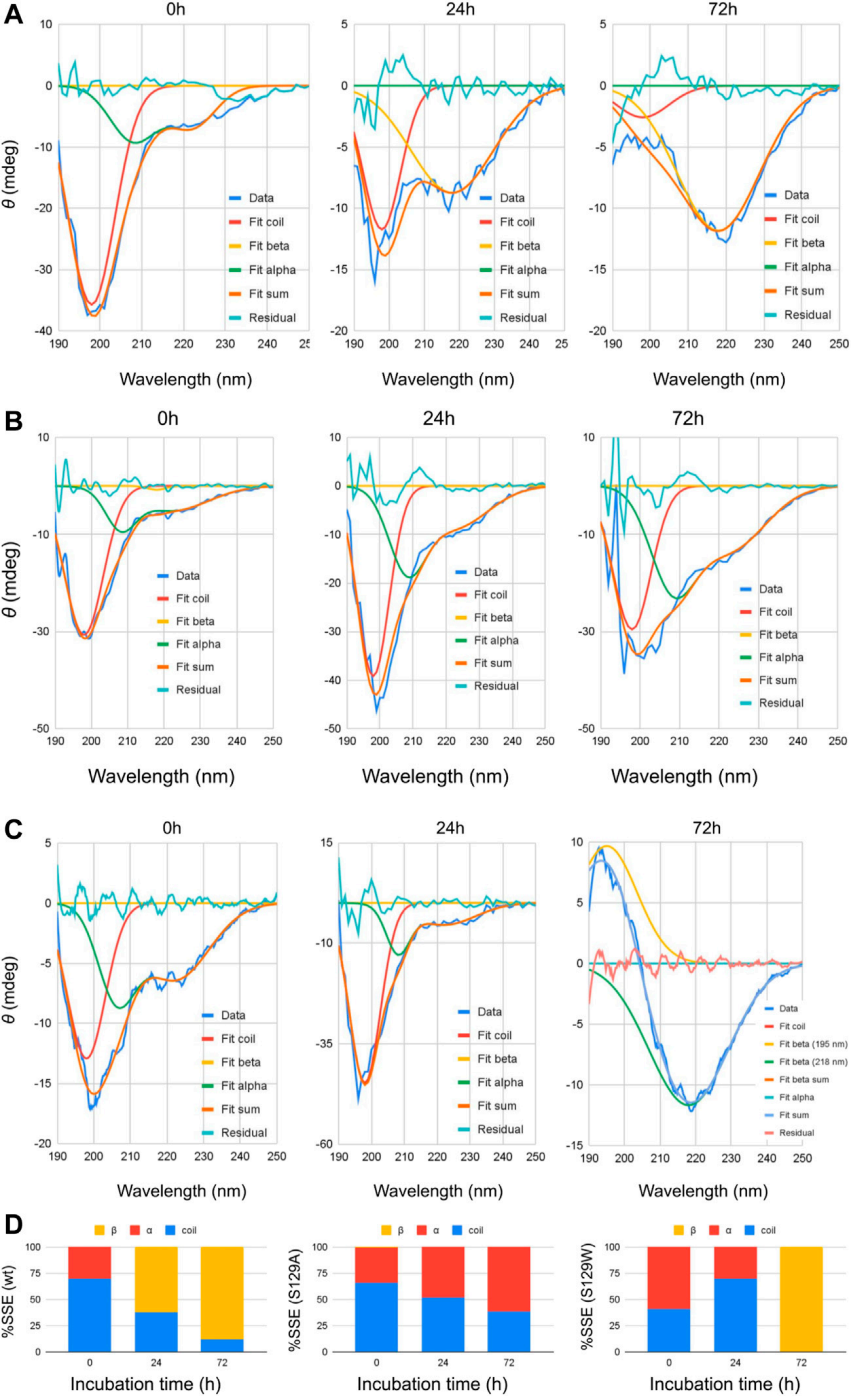
Circular dichroism spectra of wt and mutant alpha synucleins and spectral deconvolution. One Gaussian at 198 nm is fitted for random coil and one at 218 nm for beta sheets. Two Gaussians at 208 and 222 nm are fitted for alpha helix, and the sum of the two is shown as “Fit alpha.” Summation of all the fitted Gaussians and the residuals are also shown. **(A)** CD spectra of wild-type aSn protein at different time points of incubation. **(B)** Circular dichroism spectra of S129A α-synuclein at different time points of incubation. **(C)** Circular dichroism spectra of S129W α-synuclein at different time points of incubation. For 72 h, a positive peak is observed at 195 nm, which is a signature of the beta-sheet. One Gaussian is additionally fitted for the beta-sheet in this spectrum. Summation of all the fitted Gaussians and the residuals are also shown. **(D)** Changes in the secondary structural composition over the period of incubation for wt and the mutants.

Circular dichroism (CD) spectroscopic measurement is an excellent way to determine the nature of the secondary structure and unfolding/folding properties of proteins *in vitro*. Changes in CD spectra correlate any alterations of the protein secondary structure. Our CD analysis with wt aS shows a primarily random coil structure with a strong negative CD band at 198 nm (Figure 1). Deconvolution of the spectrum, however, revealed the presence of some α-helical signatures in the protein secondary structure. Computation of the area under the curves showed 31% α-helix and 69% random/disordered conformation in wt aS (Table 1). After 24 h of incubation, the random coil signature was decreased from 69% to 38%. However, the β-sheet signature became prominent (62%). After subsequent incubation for 72 h, the presence of random coil further reduced to 12% and the protein attained mostly (88%) β-sheet conformation, confirming formation of compact and well-defined β-sheet rich amyloid fibrils. The initial structure of S129A was very similar to that of the wt aS (Figure 1). However, upon incubation for 24 h, the helical signature of mutant protein increased from 34% to 48% and finally to 62% (Table 1). No major β-sheet signature was observed. This result indicated that the substitution of the serine 129 residue with alanine enhanced the propensity of helix formation in its oligomeric states. The S129W mutant in the monomeric state itself showed more α-helical patterns than the wild-type and the S129A mutants (Figure 1; Table 1). After 24 h of incubation, some decrease in the helical signature was observed; however, no β-sheet signature was apparent. However, after 72 h of incubation, the mutant attained mostly a β-sheet structure. It was also confirmed by ThT fluorescence assay, as discussed in the following section.

**TABLE 1:** Deconvolution of CD spectra.

Sample	Fitting		0 h				24 h				72 h			
	Peak	Remark	Depth	HWHM	Area	%Area	Depth	HWHM	Area	%Area	Depth	HWHM	Area	%Area
wt	198	Random	35.74	6.5	611	69	11.73	6.25	193	38	2.58	8.13	55	12
	208	Alpha	9	6.5	154	31	0	0	0	0	0	0	0	0
	222		6.76	6.5	116		0	0	0		0	0	0	
	218	Beta	0	0	0	0	8.74	13.79	317	62	11.83	12.76	397	88
S129A	198	Random	30.67	6.22	502	66	39.21	5.62	580	52	29.58	5.64	439	38
	208	Alpha	7.42	4.84	95	34	15.07	6.43	255	48	18.2	6.61	317	62
	222		5.15	11.97	162		8.75	12.32	284		13.44	11.14	394	
	218	Beta	0.79	2.96	6	1	0	0	0	0	0	0	0	0
S129W	198	Random	12.92	6.06	206	41	44.84	5.54	654	70	0	0	0	0
	208	Alpha	7.07	5.94	111	59	11.03	4.15	120	30	0	0	0	0
	222		6.4	11.06	186		5.51	11.36	165		0	0	0	
	218	Beta	0	0	0	0	0	0	0	0	11.68	13.17	405	100

### Aggregation kinetics of α-synucleins by thioflavin T fluorescence assay and atomic force microscopy

**FIGURE 2:**
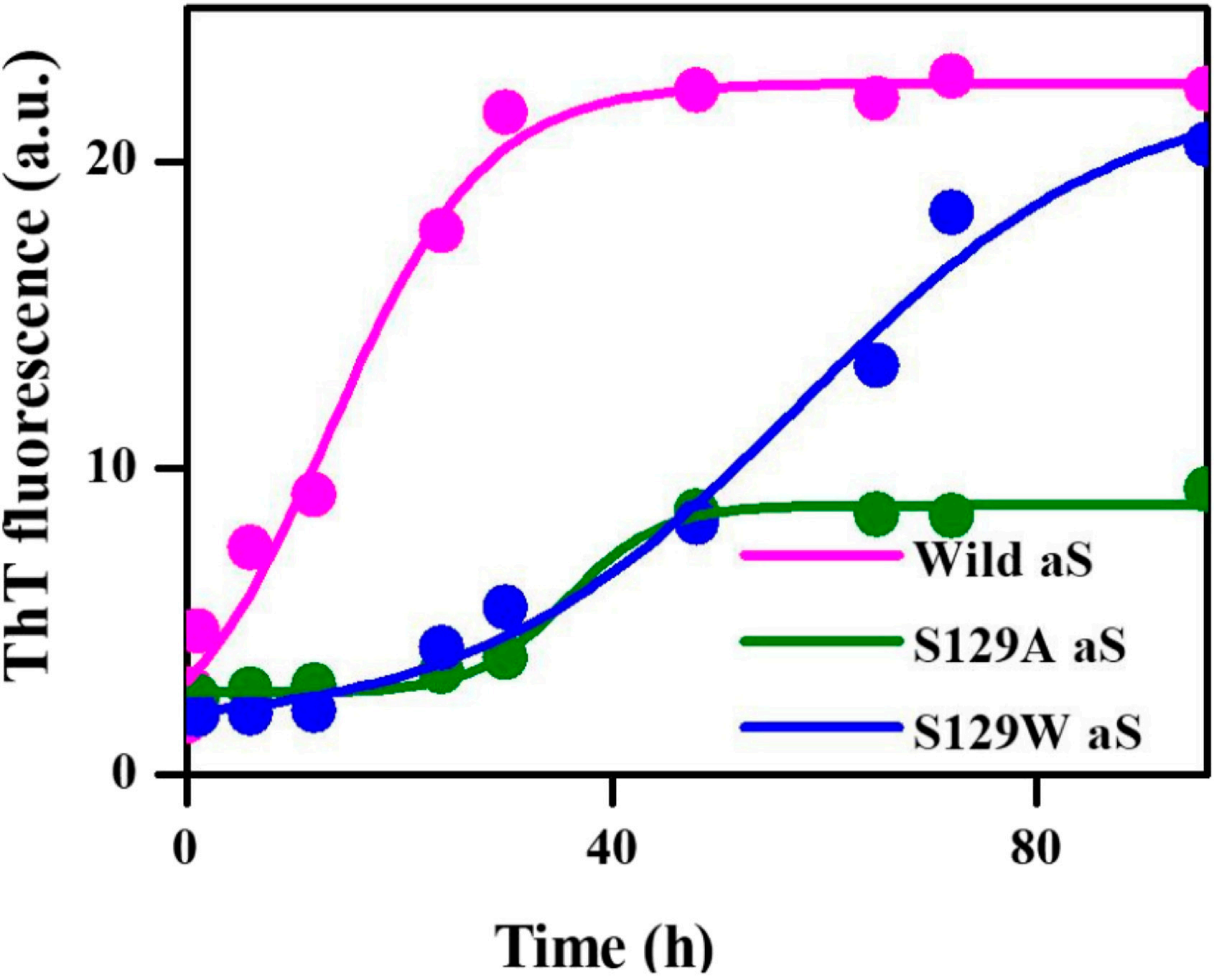
Time-dependent ThT fluorescence assay for determining the fibril formation growth kinetics of wild-type α-synuclein (wt aS) and the mutant variants (S129A and S129W). For each incubated sample, at different time points of incubation, 20 µL of the incubated solution was mixed with 500 µL of ThT solution (∼20 µM) and the ThT fluorescence was measured, and the sample was excited at 440 nm. The magenta line is the fit of Eq. 1 to the data points obtained for wt aS. Similarly, the olive line is the fit of Eq. 1 to the data points obtained for S129A, and the blue line is a fit of Eq. 1 to the data points obtained for S129W.

Thioflavin T (ThT) assay was performed to measure the fibrillization kinetics of both the mutant and wild-type aS. ThT is a fluorescence dye and is often used to monitor *in vitro* amyloid fibril formation. Upon binding to amyloid fibrils, ThT emits a strong fluorescence signal at ∼485 nm when excited at 450 nm (Voropai et al., 2003) Figure 2 shows the time-dependent ThT fluorescence assay for determining the fibril formation growth kinetics of wt aS and the mutant variants (S129A and S129W). The increment of fluorescence intensity for wt aS was faster than both the mutant aS. ThT fluorescence intensity was gradually increasing depending upon the incubation time of the protein solution, until it reached a plateau by ∼40 h. The fluorescence intensity subsequently remained steady until 90 h (Figure 2). The mechanism of the enhanced ThT fluorescence signal upon binding to β-sheet-rich fibrils has been attributed to the rotational immobilization of the central C–C bond of ThT, which connects its benzothiazole and aniline rings (Stsiapura et al., 2007; Srivastava et al., 2010). On the other hand, the lack of properly formed amyloid fibrils is indicated by weak fluorescence signals of the dye molecules present in the solution. ThT kinetics data, according to the protocol reported by Meisl et al. (2016), to understand the type of elongation as the slopes of the ThT kinetics graphs, differ significantly for the two mutant aS compared to the wild-type proteins. The wt aS showed nucleation-dependent elongation. It depends only on the concentration of free monomers. Thus, based on the fluorescence enhancement and incubation time, the fitted equation (Eq. 1) to the data suggested that fibril formation reached to its half maxima (t_1/2_) at 13.6 h; the apparent rate constant (*K*_*app*_) of fibrillation of wt aS was 0.13 h^−1^. In contrast, the ThT fluorescence, in the presence of incubated S129W, increased at a slower rate than wild-type aS (wt aS). No significant rise in ThT fluorescence was observed up to 24 h. However, after 24 h of incubation, gradual increment of ThT fluorescence was observed and it was attributed to the formation of aggregates with beta-sheet conformation. The t_1/2_ and apparent rate constant (*K*_*app*_) of fibrillation for this mutant also were measured by fitting the data points using Eq. 1, and the values were 59 and 0.06 h^−1^, respectively. Under the same conditions, S129A also showed a slow process of fibrillization. It has a prolonged lag phase and the half maxima (t_1/2_) of 35 h, and the apparent rate constant (*K*_*app*_) of fibrillation was 0.23 h^−1^.

**FIGURE 3:**
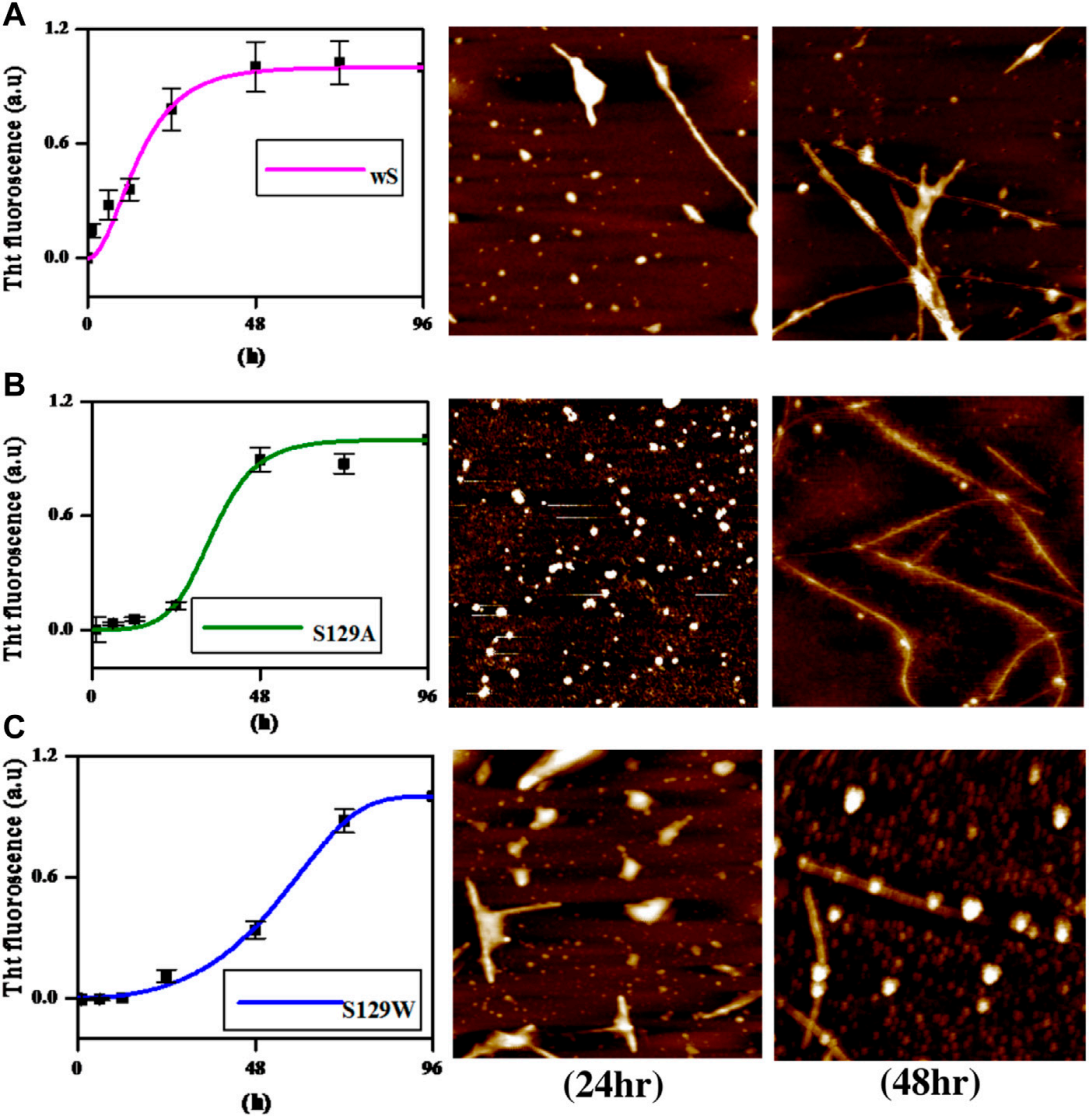
Different elongation kinetics of wt, S129A, and S129W aS proteins as obtained from AmyloFit and surface morphology of α-synuclein aggregates formed at different time points, as observed by atomic force microscopy. **(A)** Nucleation elongation kinetics for wild-type aS and AFM images obtained at 24 and 72 h. **(B)** Secondary nucleation-based elongation kinetics for S129A and AFM images obtained at 24 and 72 h. **(C)** Fragmentation-dominated elongation kinetics for S129W and AFM images obtained at 24 and 72 h. All AFM images are of dimension 2.5 µm × 2.5 µm.

AFM (atomic force microscopy) was further engaged to visualize the morphological features of the aggregates produced in the processes of fibrillation. At 24 h of incubation, the wild protein showed the formation of protofibrils along with oligomers (Figure 3A). An average mean height of spherical oligomers was ∼6.4 nm. For the same time period of incubation, S129A and S129W yielded oligomers of diameters of ∼3.8 and ∼5.0 nm, respectively. Fibrils were visible for all the three variants of proteins at 72 h of incubation. The wild protein produced a mesh-like fibrillar network with an average mean height of 6.08 nm (Figure 3A). It was also observed that both the mutants produced protofibirillar structures. The S129A mutant produced very thin fibrils (Figure 3B), and the S129W mutant showed thicker protofibrils with oligomers still visible (Figure 3C). The average mean height of fibrils produced from S129A and S129W were 5.8 and 6.3 nm, respectively, as shown in Figure 3. The average mean height of all the oligomers and fibril samples is calculated taking an average sample size between 5 and 8 (Supplementary Figure S3A, S3B; Supplementary Table S1A). The fibril lengths for each species were ∼2.5 µm, and no significant difference was observed.

### Structural features of the protein and their aggregates from the Raman spectral signature

**FIGURE 4:**
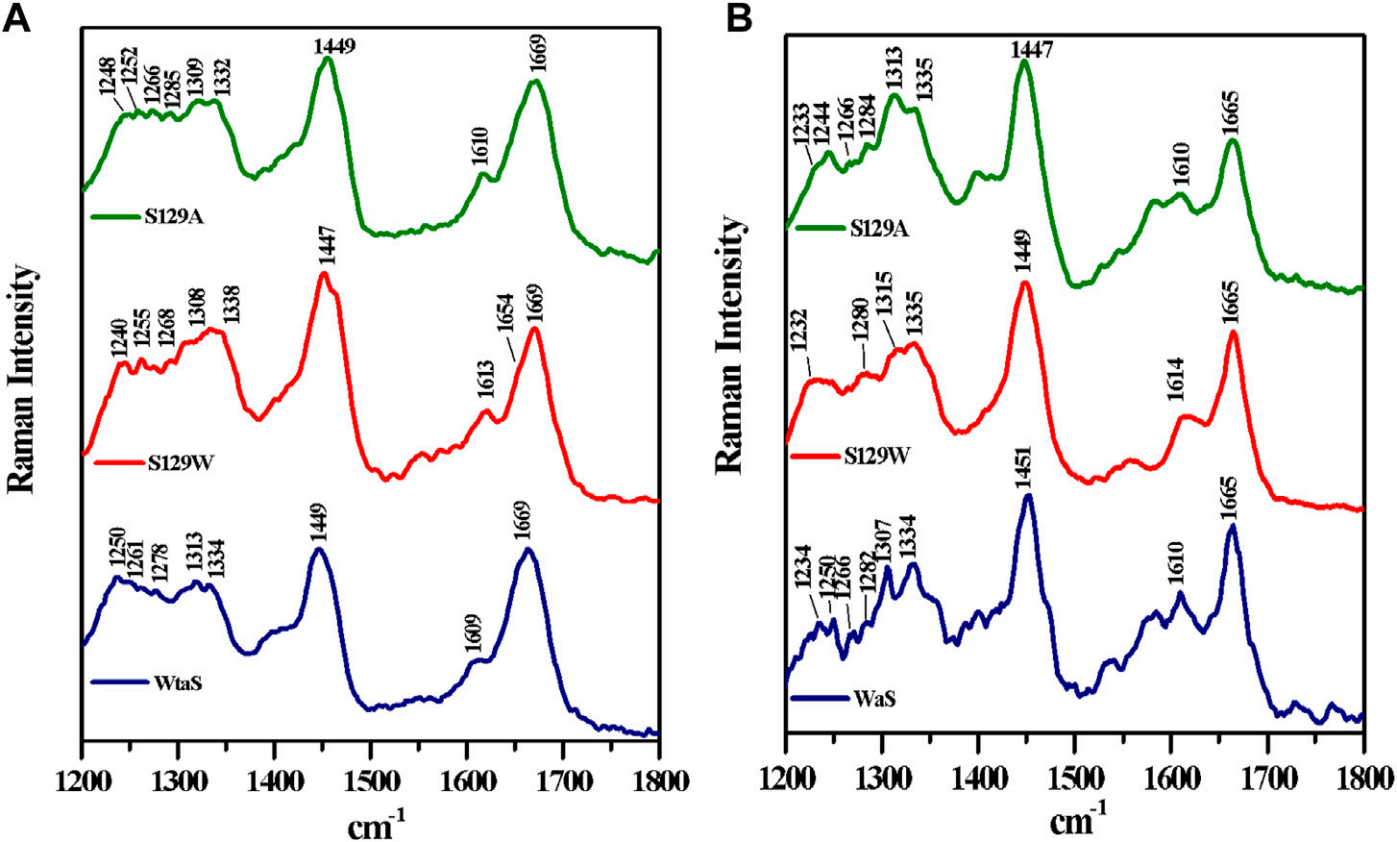
Raman spectra of wild and two mutants of alpha synuclein, their monomer, and fibril in the frequency range of 1200–1800 cm^−1^ by 532-nm laser. Both monomer **(A)** and fibril **(B)** solution were prepared in 20 mM sodium phosphate buffer pH 7.4 and dropped cast on a glass coverslip, and spectra were recorded at room temperature (25 °C) after being air dried. Laser power at the source was 50 mW at the sample.

Raman spectroscopy is used to study the structural features of proteins and peptides in different stages of aggregation and fibrillation (Apetri et al., 2006; Kurouski et al., 2015; Flynn et al., 2018). Figures 4A, B show the Raman signature of the monomer and fibril of wt aS, S129W, and S129A. The most structure-sensitive amide I band of proteins appears at 1620–1700 cm^−1^, and the amide III band is often observed at the 1200–1320 cm^−1^ frequency range. The Raman peaks of the protein samples are listed in Table 2. The Raman spectrum of freshly prepared wt aS in its fairly monomeric state showed a broad amide I band centered at 1669 cm^−1^ with a full width at half maxima (FWHM) of 55 cm^−1^. It suggested a highly fluctuating secondary structure, similar to previously reported data (Maiti et al., 2004). The amide I band became quite narrow (FWHM 37 cm^−1^), and it was from 1669 cm^−1^ to 1665 cm^−1^, which was consistent with progressive increases in the ordered secondary structure, primarily the β-sheet conformations in this case. The Raman signature of the S129A monomer is shown in Figure 4A. It also exhibited a broad amide I Raman band at 1669 cm^−1^ with an FWHM of 62 cm^−1^. It also suggested the presence of conformational heterogeneity in its secondary structure. In S129A fibrils (Figure 4B), the Raman amide I band becomes somewhat narrow (FWHM 46 cm^−1−1^) along with a shift in peak position from 1669 to 1665 cm^−1^, indicating appearance of the ordered secondary structure. The S129W monomer amide I band shows an FWHM of 53 cm^−1^, whereas in S129W fibrils, the Raman amide I band became narrow (FWHM 38 cm^−1^), and there was a shift in the band position from 1669 to 1665 cm^−1^, which also suggested the presence of ordered secondary structural conformation such as the β-sheet. However, further investigation indicated broad shoulders near 1655 cm^−1^ for both the mutants. Amide I band analysis suggested that wt aS and both the mutant variants may contain multiple secondary structures which are dynamic in nature. An exact differentiation of the structural content in the different variants of the protein was difficult. However, a broader shoulder near 1655 cm^−1^ clearly suggested presence of residues in the α-helical space. Fibrils formed from the S129A mutant showed a greater band width (FWHM 46 cm^−1^) than the width observed for wt aS and S129W. It suggested a less compact β-sheet structure of the fibrils produced from S129A. It was also reflected in less amount of ThT fluorescence at an equilibrium/steady state of condition after 72 h (Figure 3B).

**TABLE 2:** Raman vibrational bands (cm^−1^) of alpha-synuclein as the monomer and fiber states.

wt aS (monomer)	S129A aS (monomer)	S129W aS (monomer)	wt aS (fiber)	S129A aS (fiber)	S129W aS (fiber)	Modes of Raman vibration
—	—	—	1,234	—	1,232	Amide III, β-sheet
—	—	1,239	—	1,244	1,247	Amide III, random coil
1,250	1,252	1,255	1,250	—	—	Amide III, random coil
1,261	1,266	1,268	1,266	1,266	1,265	Amide III, α-helix
1,278	1,285		1,282	1,284	1,280	Amide III, α-helix
1,313	1,309	1,308	1,307	1,313	1,315	CH2 twist
1,334	1,332	1,338	1,334	1,335	1,335	CH2 twist
1,449	1,449	1,447	1,451	1,447	1,449	CH2, CH3 deformation
1,609	1,610	1,613	1,610	1,610	1,614	Tyr
—	—		1,665	1,665	1,665	Amide I, β-sheet
1,669	1,669	1,669	—	—	—	Amide I, random coil

The amide III region (1200–1320 cm^−1^) also provides some signature of the protein conformations. The band originated due to in-phase N–H deformation and C–N stretching. Recent articles by Dolui et al. and references therein provide the details of band positions. All the three variants of aS (wt aS, S129A, and S12W) showed a weak band at ∼1252 cm^−1^ (precisely for wt aS, the band was at 1250 cm^−1^, for S129W 1255 and 1252 cm^−1^ for S129A) The amide III band near 1250 cm^−1^ is assigned to mainly disordered or poly-L-proline conformation (Dolui et al., 2020 and the references therein). The shoulder band position near 1278 and 1261 cm^−1^ sometimes indicated the presence of α-helical conformation. These bands were present with slight variation in all the three forms of the monomeric proteins (Figure 4A). Upon fibril formation, the β-sheet signature band appeared near 1234 cm^−1^; however, the band position for S129A was much weaker. However, the weak intensity and contribution of vibrational bands originating from the side chain of amino acid residues in this region render precise structural component analysis difficult and it was avoided.

### Cytotoxicity evaluation of wild and mutated alpha synuclein using MTT assay

**FIGURE 5:**
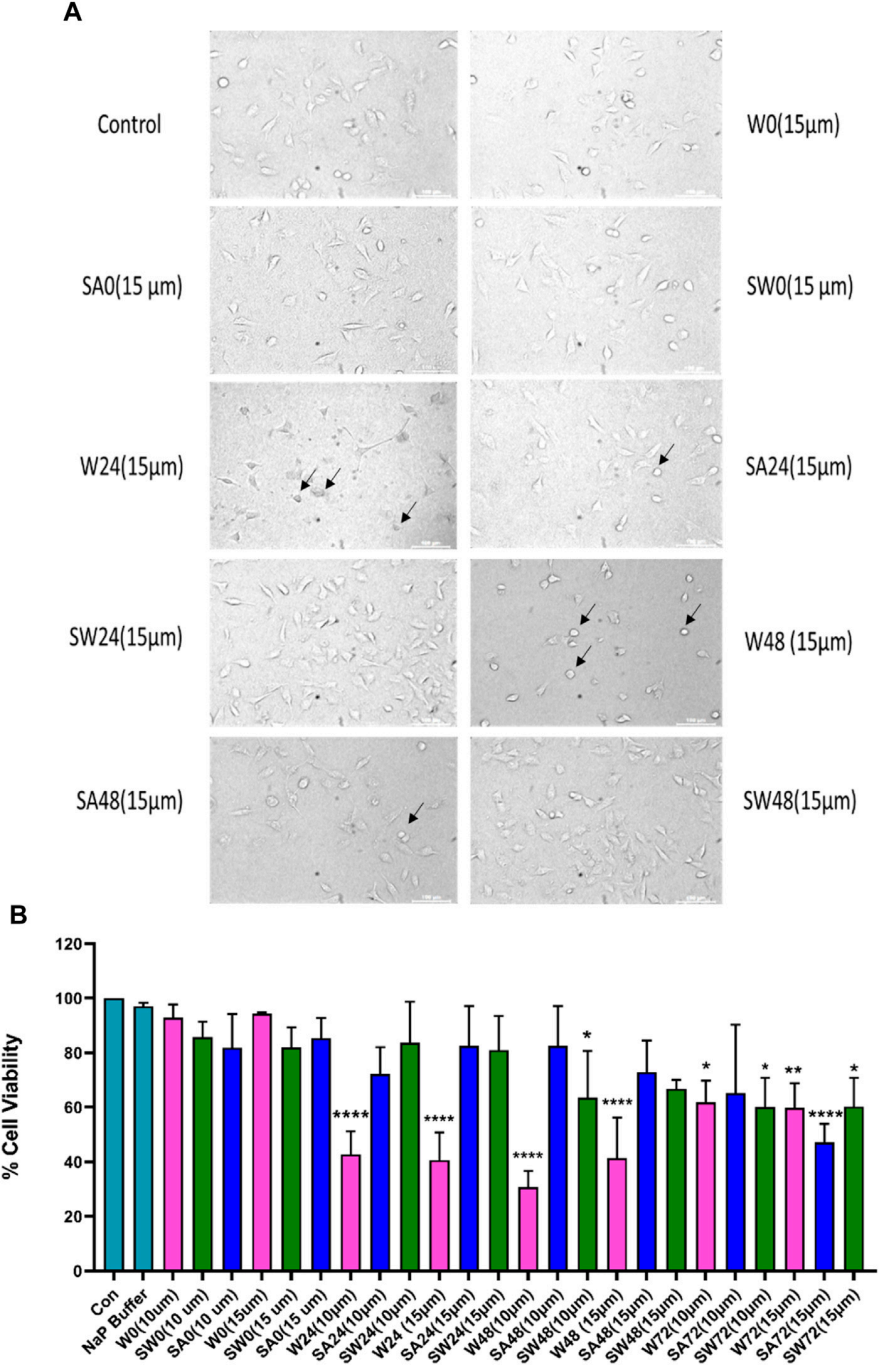
Cytotoxicity evaluation of wild-type and mutant aS proteins using MTT assay. Wild-type and mutant protein oligomers were incubated for 0, 24, 48, and 72 h, and then, their effects were checked for viability of SH-SY5Y neuroblastoma cells. **(A)** Representative microscopic images of SH-SY5Y cells after 24 h treatment of different variants of wild-type and mutant proteins. Cells were treated with 15 µM concentration for imaging purposes. Arrows indicate dead cells. **(B)** Cells were treated with two different concentrations of oligomer variants (10 μM and 15 µM) and incubated for 24 h, followed by MTT assay. All experiments were performed in triplicate, and the relative cell viability (%) was expressed as a percentage relative to the untreated cell (control) as mean ± SEM in a bar diagram where *, *p* ≤ 0.05; **, *p* ≤ 0.01; ***, *p* ≤ 0.001; ****, *p* ≤ 0.0001 compared to control. NaP, sodium phosphate buffer; W0, W24, W48, and W72, wild-type alpha synuclein incubated for 0, 24, 48, and 72 h, respectively; SW0, SW24, SW48, and SW72 mutant S129W alpha-synuclein incubated for 0, 24, 48, and 72 h, respectively; SA0, SA24, SA48, and SA72 mutant S129A alpha-synuclein incubated for 0, 24, 48, and 72 h, respectively.

**FIGURE 6:**
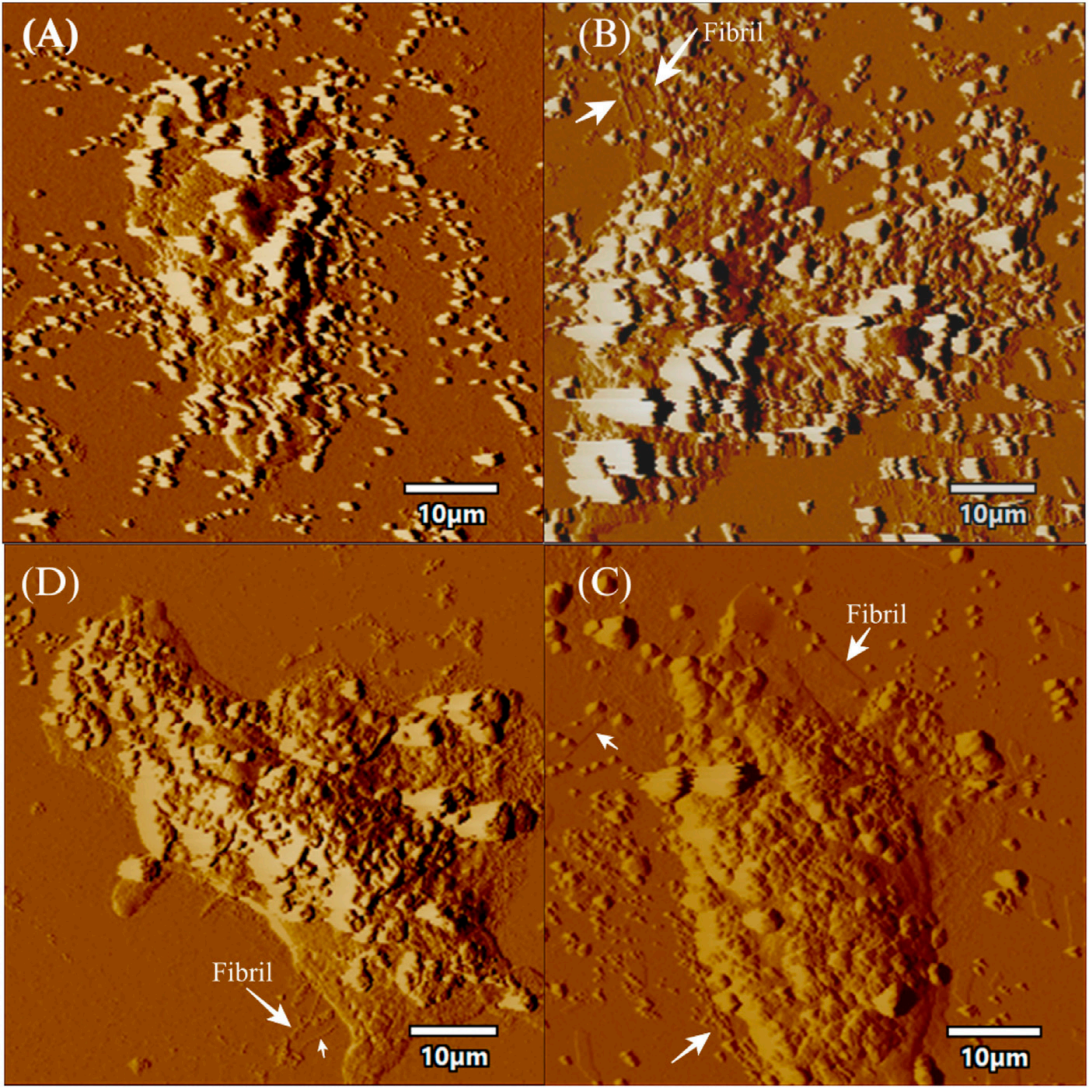
AFM topographic images of SH-SY5Y cells in presence of fibrils. The figure displays topographic images acquired through atomic force microscopy (AFM) of SH-SY5Y cells. SH-SY5Y cells were incubated for 24 h using 72 h incubated aS. Then, the cells were processed for AFM, which **(A)** depicts control cells without any alpha-synuclein (aS) fibrils, while **(B)** shows SH-SY5Y cells in the presence of wild-type aS fibrils. **(C)** shows SH-SY5Y cells exposed to the S129A aS fibril, and **(D)** displays SH-SY5Y cells exposed to the S129W aS fibril. In all panels, the presence of fibrils is indicated with a white arrow. Scale bar = 10 µm.

Studying the cell viability of SH-SY5Y cells upon exposure to different aS species has been extensively used in recent times (Mahapatra et al., 2019). It helps in accessing the toxicity of aggregates formed at different time points of incubation. It has been shown that extracellular aS fibrils are very often transported to neurons and play a significant role in aS pathology (Honarmand et al., 2019). Adding incubated samples of aS aggregates to neural cells helps in mimicking the prion-like nature of aggregated aS protein (Brundin et al., 2010). Several studies have also reported that aS oligomers and intermediates formed during aggregation are more toxic than fibrillar structures (Winner et al., 2011). Intrigued by that information, we investigated the cytotoxic effect of aggregates of wt aS and S129A aS and S129W aS in neuronal SH-SY5Y cells. The cells were treated with the oligomers, protofibrils, and fibers of different concentration in a time-dependent manner. Cells treated with early forms of aggregates formed by wild-type protein showed a considerably higher level of cytotoxicity than mutant variants (Figures 5A, B; Table 3). With respect to control, cells treated with wild-type protein aggregates collected after 24 and 48 h of incubation showed significant amount of cell death compared to cells treated with aggregates of mutated proteins collected from a similar time point of incubation, which showed much higher survivability. Cell death for wt aS-treated samples is highest when aggregates formed after 24 h of incubation are used to treat the cells (only ∼40% cells survived compared to ∼80% in case of both mutant proteins). Cells treated with aggregates formed by mutated variants obtained after 24 h of incubation show negligible cell death. The MTT assay results thus indicate that S129A and S129W point mutations in aS make it less toxic toward neuronal cells probably by reducing oligomer formation due to imparted stability of the monomeric states. In addition, it is interesting to see how the aggregates formed after 72 h of incubation are affecting the cell’s survivability. In case of wild-type protein, after 72 h of incubation, the percentage of cell survivability goes up (∼60% compared to ∼40% cell survival seen in case of treatment with oligomers.) This aligns with the previous finding of fibrils being less toxic than oligomers. We can also conclude that in the case of wild aS, most oligomers form protofibrils and fibrils after 72 h of incubation, which is also confirmed by ThT and AFM data. However, in case of mutant proteins, the cell survivability decreases when treatment is performed with samples collected after 72 h of incubation (∼60% in case of both mutant proteins S129A and S129W), which is significantly less than treatments performed with samples collected from 24 to 48 h of incubation. Cells incubated for 24 h with aggregated samples (prepared after 72 h of incubation) were also observed under AFM (Figure 6). This indicates that there was a probability that these fibrils were more toxic in nature, which leaves scope for further studies.

**TABLE 3:** Average cell survivability rate of SH-SY5Y cells when treated with different concentrations of oligomers formed by wt aS and S129A aS and S129W aS collected at different time points of incubation.

Treatment used	Average
Control	100
Sodium phosphate buffer	97.03
wt aS (0 h, 10 µM)	92.9
wt aS (0 h, 15 µM)	94.3
S129A aS (0 h, 10 µM)	81.8
S129A aS (0 h, 15 µM)	85.3
S129W aS (0 h, 10 µM)	85.7
S129W aS (0 h, 15 µM)	81.9
wt aS (24 h, 10 µM)	42.67
wt aS (24 h, 15 µM)	40.62
S129A aS (24 h, 10 µM)	72.2
S129A aS (24 h, 15 µM)	82.48
S129W aS (24 h, 10 µM)	83.6
S129W aS (24 h, 15 µM)	80.94
wt aS (48 h, 10 µM)	30.77
wt aS (48 h, 15 µM)	41.46
S129A aS (48 h, 10 µM)	82.5
S129A aS (48 h, 15 µM)	72.83
S129W aS (48 h, 10 µM)	63.4
S129W aS (48 h, 15 µM)	66.8
wt aS (72 h, 10 µM)	61.7
wt aS (72 h, 15 µM)	59.8
S129A aS (72 h, 10 µM)	65
S129A aS (72 h, 15 µM)	41
S129W aS (72 h, 10 µM)	60
S129W aS (72 h, 15 µM)	60

## Conclusion

Our current investigation established how single point mutation, particularly in the highly dynamic C-terminal region, makes profound impacts on the aS structure, dynamics, and formation-specific aggregates of distinct morphologies. The study was focused on the S129 sequence position in the C-terminal soluble domain of aS. We found that replacement of this hydrophilic serine residue with alanine (considered as the most helix promoting residue), which is eventually a replacement of the polar side chain with a non-polar methyl group, reduced the aggregation propensity of aS significantly and hindered the formation of a very compact β-sheet structure. Similarly, replacing the serine residue with hydrophobic tryptophan (has both helix and β-sheet propensity) affects the aggregation processes; the lag phase of aggregation lengthened compared to wt aS. It was, thus, established that increased hydrophobicity/helix-forming propensity in the C-terminal domain provided stability to the monomeric form of the protein, slowing down both oligomerization and subsequent fiber formation processes. ThT fluorescence assay CD and Raman spectral analysis all suggested β-sheet-rich compact fibrils for wt aS and S129W after 72 h of incubation, although β-sheets may not be very compact for S129A due to the presence of alanine, a helix promoting residue. The results, thus, established that mutation of an important residue in the soluble tail of aS with the helix-promoting residue reduced its aggregation and neurotoxic effect on neuronal cells.

## Materials and methods

### Expression and purification of wild-type and mutant α-synuclein

The plasmid containing aS gene was gifted by Hilal Lashuel (Addgene plasmid # 36046). aS was overexpressed in *E. coli* BL21 (DE3) using plasmid pT7-7 encoded with wild-type human aS gene, following a previously published protocol. For the S129A and S129W mutated variants, the vector was constructed by replacing the codon AGT for serine 129 with GCC, which encodes alanine, and TGG, which encodes tryptophan (obtained from GenScript). Overexpression of mutant proteins was conducted in *E. coli* BL21 (DE3), following a similar protocol as the wild-type protein. Bacterial cells were cultured overnight at 37 °C in Luria Broth in the presence of ampicillin (100 μg/mL) as a selection agent. Overnight primary culture was followed by a secondary culture in LB under the same conditions. Once the optical density (OD) of the secondary culture reached 0.6, 1 mM isopropyl-β-D-thiogalactopyranoside (IPTG) was added for the induction of the protein overexpression. Bacterial pellets were harvested by centrifugation at 10000 rpm for 15 min at 4 °C. The pellets were resuspended in a TEN buffer composed of 30 mM Tris pH 7.5, 10 mM ethylenediaminetetraacetate, and 100 mM NaCl along with 1 mM phenylmethanesulfonyl fluoride for cell lysis. The lysate was then heated at 75 °C for 10 min and then centrifuged at 18000 rpm, and the supernatant enriched in aS was collected. The supernatant was transferred to a fresh tube, and streptomycin sulfate (136 μL of 10% solution/mL of the supernatant) and acetic acid (glacial, 228 μL/mL of supernatant) were added to it, followed by another centrifugation for 15 min. aS was then purified by fractional precipitation. Established protocols for the precipitation method were followed for protein purification for both wt aS and mutated aS variants.^54^ The supernatant was subjected to ammonium sulfate precipitation (4 °C, 1:1 v/v saturated ammonium sulfate: water) and centrifuged for an hour. The pellet was collected and washed with 2 mL of 50% ammonium sulfate solution. The washed pellet was resuspended in 100 mM ammonium acetate solution, which was added gradually until a cloudy solution was formed, followed by ethanol precipitation. Ethanol precipitation was conducted once more followed by a final resuspension in 100 mM ammonium acetate solution. Overnight dialysis was performed in water at 4 °C, followed by freezing and lyophilization. The purity of the protein was checked using SDS-PAGE and MALDI analysis (S2A, S2B).

### Induction of aggregation *in vitro*

To obtain oligomers and fibrils, lyophilized protein was dissolved in a 20 mM phosphate buffer (pH 7.4), and the concentration was adjusted by measuring UV absorption at 276 nm. Using the known extinction coefficient of 5960 M^−1^cm^−1^ at 276 nm for pure aS, a final concentration of 350 µM protein was prepared for all the samples that were set for incubation. The samples were incubated under constant shaking 700 rpm at 37 °C in a BR Biochem Thermo Mixer (BITH-100). Aliquots of incubated samples of wS and mS were extracted at certain intervals of incubation for spectroscopic and microscopic analyses.

### Circular dichroism spectroscopy

The far-UV circular dichroism (CD) spectra (190–250 nm) were recorded on a JASCO J-815 spectropolarimeter (Jasco) equipped with a Peltier temperature control unit, using a 1-mm path length cuvette. All of the CD measurements were performed at room temperature (∼25 °C), and the buffer background was subtracted. For each sample, three scans were averaged. Then, 350 μM aS stock was prepared by dissolving the lyophilized powder in a 20 mM sodium phosphate buffer with 0.01% sodium azide solution and incubated under constant shaking (700 rpm) at 37 °C. For the CD analysis, prior to spectral acquisition, 15 µL of the stock solution was dissolved in 500 µL of 20 mM sodium phosphate buffers to get a diluted solution of aS with a final concentration of ∼10 µM.

Spectral deconvolution was performed by fitting Gaussians at the known peak positions for random coil (189 nm, negative band), alpha helix (208 and 222 nm, negative bands), and beta sheet (218 nm, negative band) using Fityk curve fitting and peak fitting software, and the peak depth/height and half width at half maximum (HWHM) for the fitted Gaussians are listed in Table 1. Area under the curves was computed using the equation: Depth*FWHM/0.76, where FWHM = 2*HWHM.

### Thioflavin T fluorescence assay

For ThT fluorescence measurement, a stock solution of 5 mM ThT was prepared in 20 mM sodium phosphate buffer (pH 7.4) and fluorescence measurements were performed using a 10-mm path length quartz cuvette. To measure aggregation kinetics, aliquots of the incubated samples were extracted at certain intervals and diluted with 20 mM sodium phosphate buffer pH 7.4 to a final concentration of 3 µM. The ThT solution was added from 5 mM stock to this oligomer solution to obtain a final concentration of 20 µM ThT. The sample was then mixed thoroughly for acquisition of fluorescence emission spectra. Fluorescence spectra over the 450–560 nm wavelength range were obtained using the PTI fluorescence spectrophotometer with the excitation monochromator set at 440 nm and excitation/emission slits at 5 nm. The averages of three consecutive readings were considered for each sample. The ThT fluorescence peak intensity at 482 nm was plotted against incubation time of the aS samples, and the following equation (Eq. 1) was fitted to the data to analyze aggregation kinetics:

$$F\left(t\right)=F_{\max}/\left(1+\mathrm{Exp}\left(-k\left(t-t_{1/2}\right)\right)\right),$$ (1)

where F is the ThT fluorescence intensity at time t, t_1/2_ is the lag time, k is the apparent rate constant, and F_max_ is the maximum fluorescence intensity. k, t_1/2_, and F_max_ are obtained by fitting the equation to F vs. t data.

Further analysis of the slopes of ThT kinetics data was conducted using the Amylofit^58^ webserver (https://amylofit.com/).

### Atomic force microscopy imaging

For the atomic force microscopy (AFM) study, aliquots were taken from the incubated samples after 24 and 48 h of incubation and diluted to 3.5 µM with distilled water. The diluted sample was then drop-casted on a freshly cleaned muscovite mica substrate and kept at room temperature for drying. Upon drying, the sample was imaged immediately with a tapping mode of AFM using a Pico plus 5500 AFM (Agilent Technologies, United States) with a piezo scanner having a maximum range of 9 µm. The images were captured with a scan speed of 0.5 line/sec. For image processing, PicoView software was used. The images of cells treated with fibrils were taken using Asylum Research MFP-3D-BIO AFM (Oxford Instruments).

### Raman spectroscopy

Raman spectra were recorded using a CTR-300 Raman spectrometer (model from Technos Instruments), which was fitted with a Nikon microscope and 300-mm focal length triple grating monochromator associated with CCD. The grating used was 600 grooves/mm. Then, 20 μL of the incubated protein samples was placed as a droplet onto a glass coverslip and air dried to obtain spectra. A ∼50-mW (at source) argon ion laser (532 nm) was focused on the protein-enriched droplet using a ×20 microscope objective (NA = 0.4) of the microscope on the sample. No damage or spectral modifications were observed in the samples under these ambient conditions. The Raman vibrational modes’ wavenumbers were calibrated with 520 cm^−1^ the of silica wafer focused under the ×20 objective lens, and spectral resolution was found to be ∼1 cm^−1^. Spectra were recorded over 370–4000 cm^−1^ with a typical accumulation time of 100 s. Spectra were processed with GRAMS/A1 software.

### Cell viability assay (MTT assay)

SH-SY5Y is a human-derived cell line sub-cloned from the original cell line named SK-N-SH. The SH-SY5Y neuron model was created from the original SK-N-SH neuroblastoma cell phenotype, which came from a bone marrow biopsy sample of a patient who was 4 years old at the time of capture. The cell line was obtained from NCL, Pune. Cell culture media was prepared using Dulbecco’s modified Eagle’s medium (DMEM) with 10% fetal bovine serum (FBS) and 1% penicillin–streptomycin (PS). The cells were cultured in an incubator with 5% carbon dioxide at 37 °C. Then, the cells were processed for cell viability assay. The cell viability assay was carried out using the principle of reduction of 3-(4,5-dimethylthiazol-2-yl)-2,5-diphenyltetrazolium bromide (MTT) by viable cells. For this, the cells were seeded in 96-well plates at 60,000 cells/well and maintained in 200 μL of DMEM supplemented with 10% FBS and 1% PS for 24 h at 37 °C. The cells were treated with different concentrations of monomers/aggregated species. After treatment, the cells were incubated for 24 h at 37 °C and 5% CO_2_, followed by the MTT reduction assay, which was performed as previously described (Banerji et al., 2013). 6-OHDA (6-hydroxydopamine) is used as positive control as it is a standard chemical used in Parkinson’s model. All experiments were performed in triplicate, and the relative cell viability (%) was expressed as a percentage relative to the untreated cell control. Statistical data analysis was performed using one-way ANOVA analysis (Tukey’s *post hoc* test) in the GraphPad Prism 9 software, where ns, non-significant; *, *p* ≤ 0.05; **, *p* ≤ 0.01; ***, *p* ≤ 0.001; ****, *p* ≤ 0.0001.

## Data Availability

The raw data supporting the conclusion of this article will be made available by the authors, without undue reservation.
