# Supplementary material for: Single point mutations at the S129 residue of α-synuclein and their effect on structure, aggregation, and neurotoxicity
Source: Front Chem. 2023 May 26;11:1145877. doi: 10.3389/fchem.2023.1145877 (PMC10250651; doi:10.3389/fchem.2023.1145877)
Supplement: Supplementary file 1 [file DataSheet1.DOCX]

**Supporting Information for:**

**Single Point Mutations at S129 Residue of α-Synuclein and Their Effect on Structure, Aggregation and Neurotoxicity**

*Esha Pandit^1,#^, Lopamudra Das^1,#^, Anoy Kumar Das^2^, Sandip Dolui^1^, Saumen Saha^1^, Uttam Pal^1^, Animesh Mondal^1^, Joydeep Chowdhury^3^, Subhas C Biswas^2^ and Nakul C Maiti^1,^**

^1^Structural Biology and Bioinformatics Division, Indian Institute of Chemical Biology, Council of Scientific and Industrial Research, 4, Raja S.C. Mullick Road, Kolkata 700032, India

^2^Cell Biology and Physiology Division, CSIR-Indian Institute of Chemical Biology, 4 Raja S. C. Mullick Road, Kolkata 700 032, India.

^3^Department of Physics, Jadavpur University, 188, Raja S.C. Mallick Rd, Kolkata 700032, India.

^#^Contributed equally. Should be considered as co-first authors.

** Address correspondence to Nakul C. Maiti, Division of Structural Biology and Bioinformatics, CSIR-Indian Institute of Chemical Biology, 4, Raja S.C. Mullick Road, Kolkata 700032*

*E-mail: ncmaiti@iicb.res.in*

*Phone: +91-33-2499-5940*


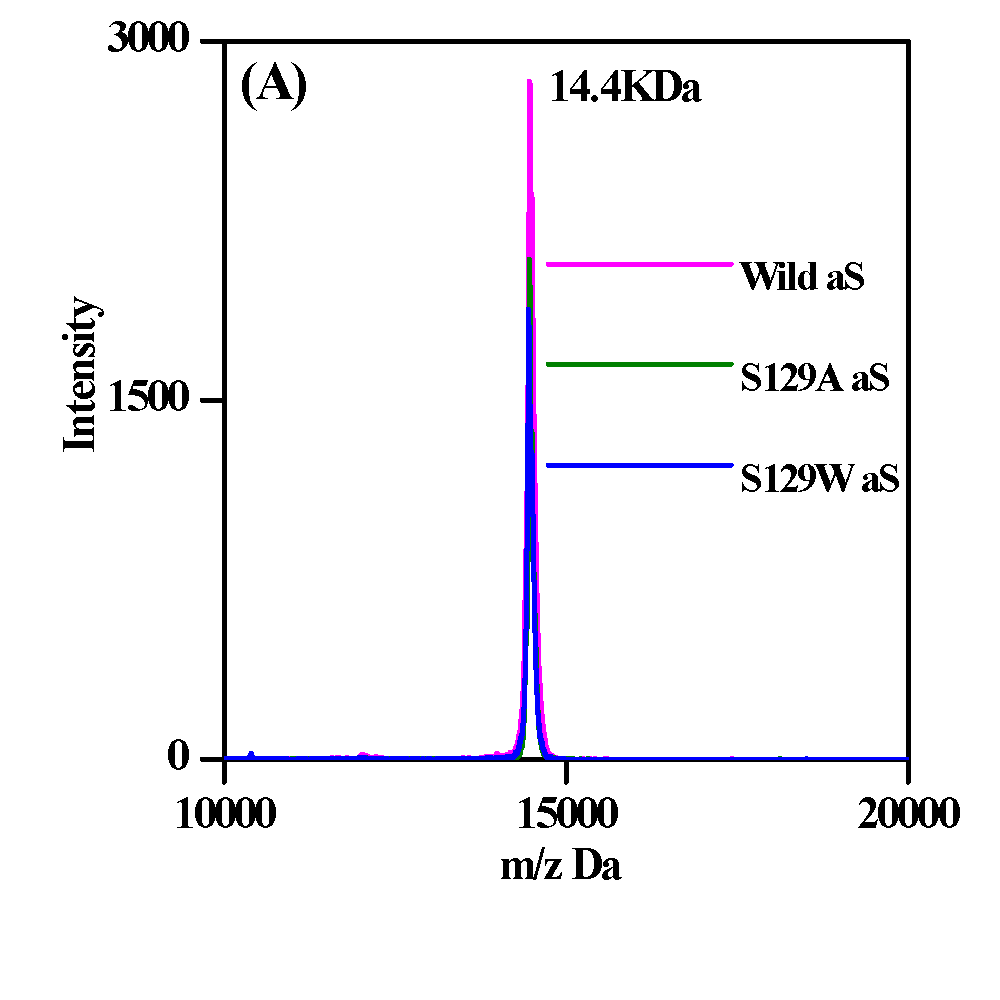

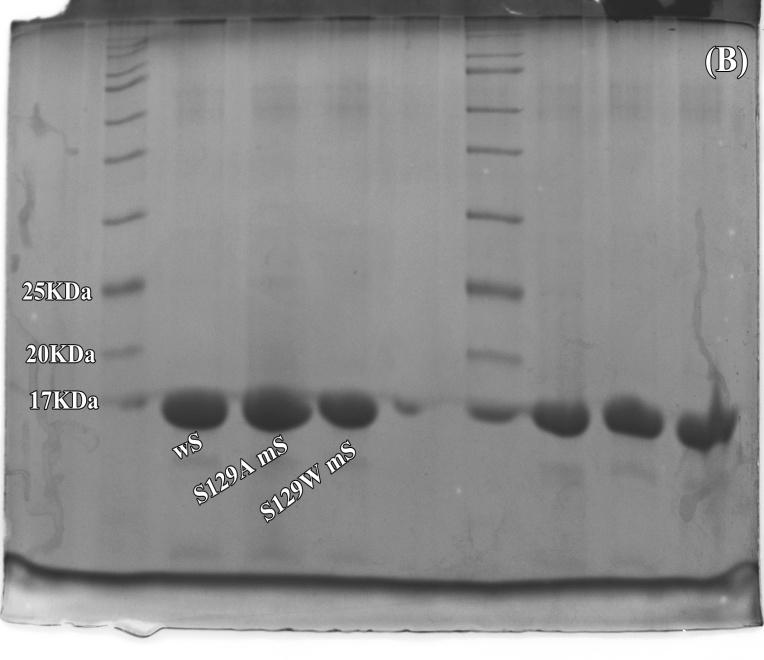


**Figure S1:** Purity data of wild type and mutant α-synucleins. (A) mass-spectrometry data. (B) SDS page gel data suggest presence of a ~14 kDa protein in monomeric form for wild type aS and the two mutant variants of aS. The first lane shows the molecular weight ladder. The following lanes indicate the proteins in the following order, wild type α-synuclein, S129A α-synuclein and S129W α-synuclein. Same sequence was repeated for ladder and protein in lanes 6,7,8, and 9 .


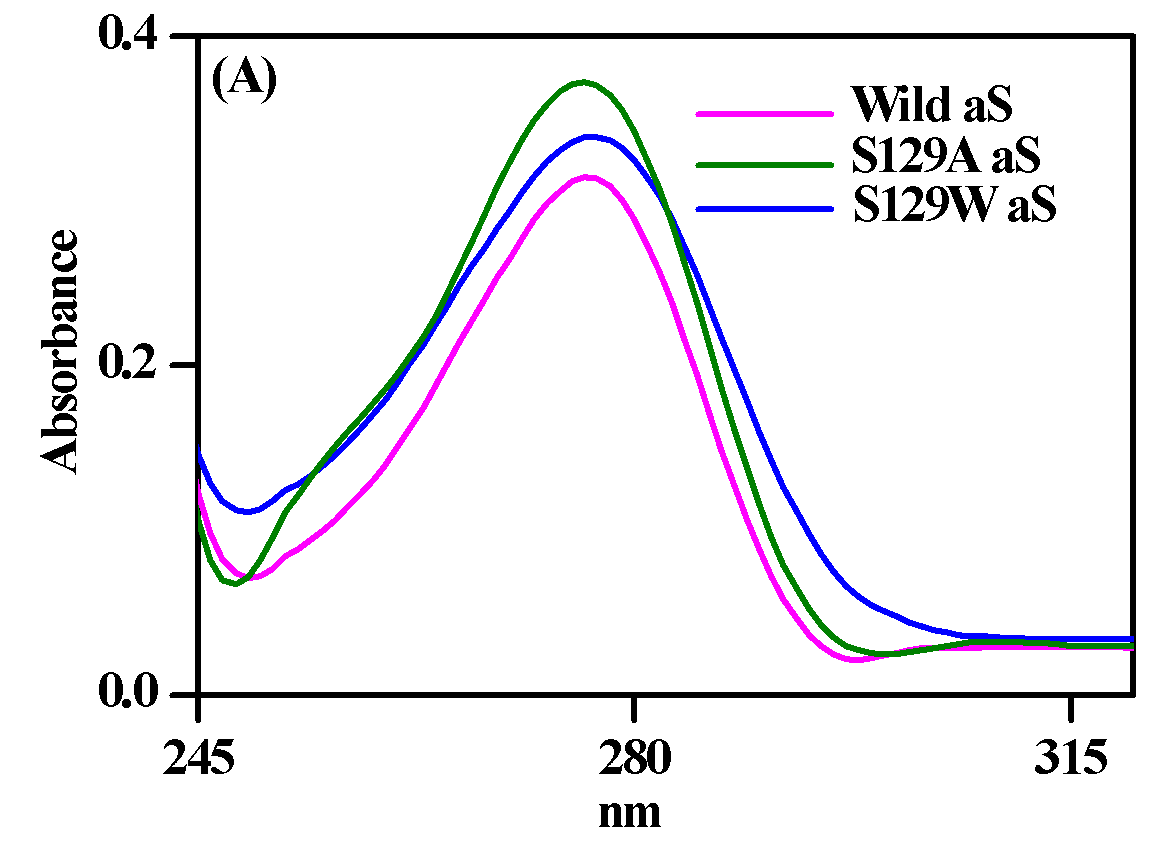

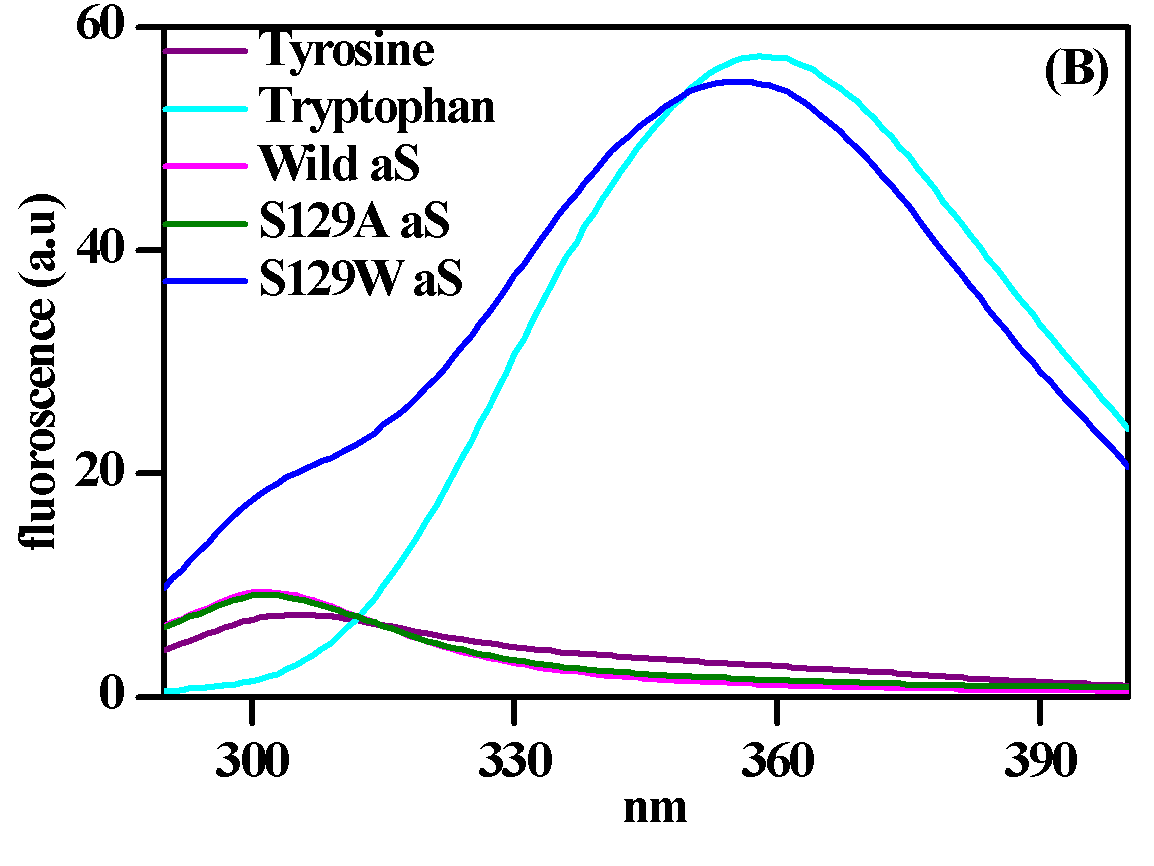


**Figure S2:** Absorption and fluorescence spectra of wild type and mutant aS. (A) Absorbance spectra of wS (magenta), S129A mS (olive) and S129W mS (blue). (B) Fluorescence spectra of wild type aS (magenta), S129A (olive) and S129W (blue). The figure also shows the fluorescence spectra of L-tyrosine (purple) and L-tryptophan (cyan). The protein solutions were prepared in a 20 mM phosphate buffer solution (pH 7.4). The concentration of Protein and amino acids was ~20µM. The excitation wavelength (λ_ex_) was276 nm for L-tyrosine and wild type and S129A aS.λ_ex_= 285nm for L-tryptophan and S129W. Both the excitation and emission slit widths were kept at 5 nm each. Spectra were recorded at room temperature (~25º)


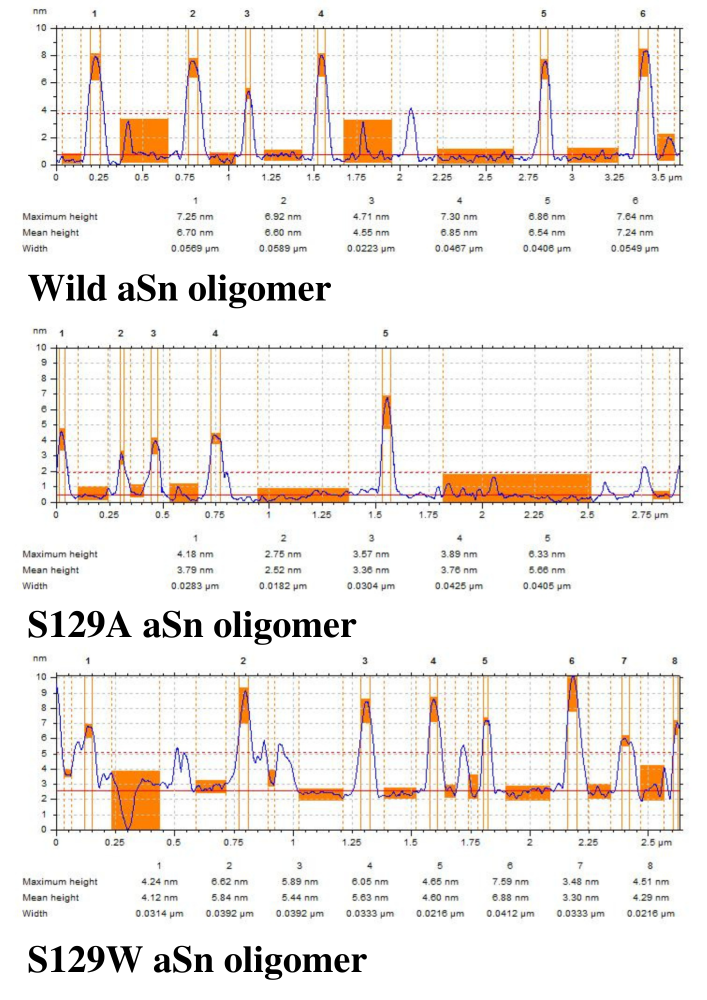

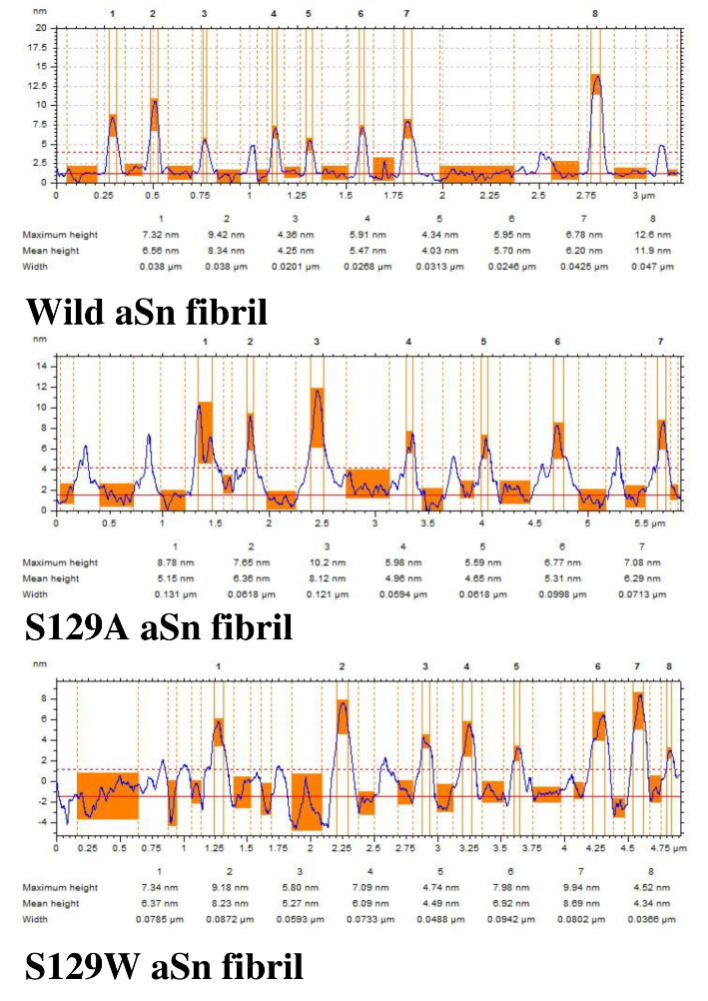


**Figure S3:** Height of oligomer and fibrils of wt, S129A and S129W aS as calculated using Picoview Software

**Table S1: Average mean height calculation of oligomers and fibrils from AFM images**

| **Sl no. \ Type** | **wt aS oligomer** | **S129A aS oligomer** | **S129W aS oligomer** | **wt aS Fiber** | **S129A aS Fiber** | **S129W aS Fiber** |
| --- | --- | --- | --- | --- | --- | --- |
| 1 | 6.7 | 3.79 | 4.12 | 6.56 | 5.15 | 6.37 |
| 2 | 6.6 | 2.52 | 5.84 | 8.34 | 6.36 | 8.23 |
| 3 | 4.55 | 3.36 | 5.44 | 4.35 | 8.12 | 5.27 |
| 4 | 6.85 | 3.76 | 5.63 | 5.47 | 4.96 | 6.09 |
| 5 | 6.54 | 5.66 | 4.6 | 4.03 | 4.65 | 4.49 |
| 6 | 7.24 |  | 6.88 | 5.7 | 5.31 | 6.92 |
| 7 |  |  | 3.3 | 6.2 | 6.29 | 8.69 |
| 8 |  |  | 4.29 | 11.9 |  | 4.34 |
| Average | 6.41 | 3.82 | 5.01 | 6.57 | 5.83 | 6.3 |
